# Fused Deposition Modelling 3D-Printed Gastro-Retentive Floating Device for Propranolol Hcl Tablets

**DOI:** 10.3390/polym15173554

**Published:** 2023-08-26

**Authors:** Abdulsalam A. Alqahtani, Abdul Aleem Mohammed, Farhat Fatima, Mohammed Muqtader Ahmed

**Affiliations:** 1 Department of Pharmaceutics, College of Pharmacy, Najran University, Najran 11001, Saudi Arabia; 2 Department of Pharmaceutics, College of Pharmacy, Prince Sattam bin Abdulaziz University, Al-Kharj 11942, Saudi Arabia

**Keywords:** 3D printing, fused deposition modelling, gastro-retentive floating device, PVA, PLA, dissolution kinetics

## Abstract

Three-dimensional printing has revolutionized drug manufacturing and has provided a solution to the limitations associated with the conventional manufacturing method by designing complex drug delivery systems with customized drug release profiles for personalized therapies. The present investigation aims to design a gastric floating tablet with prolonged gastric floating time and sustained drug release profile. In the present study, a gastro retentive floating device (GRFD) was designed and fabricated using a fused deposition modelling (FDM)-based 3D printing technique. This device acts as a multifunctional dosage form exhibiting prolonged gastric retention time and sustained drug release profile with improved oral bioavailability in the upper gastrointestinal tract. Commercial polyvinyl alcohol (PVA) and polylactic acid (PLA) filaments were used to design GRFD, which was comprised of dual compartments. The outer sealed compartment acts as an air-filled chamber that imparts buoyancy to the device and the inner compartment is filled with a commercial propranolol hydrochloride immediate-release tablet. The device is designed as a round-shaped shell with a central opening of varying size (1 mm, 2 mm, 3 mm, and 4 mm), which acts as a drug release window. Scanning electron microscope (SEM) images were used to determine morphological characterization. The in vitro buoyancy and drug release were evaluated using the USP type II dissolution apparatus. All the designed GRFDs exhibit good floating ability and sustained drug release profiles. GRFDs fabricated using PLA filament show maximum buoyancy (>24 h) and sustained drug release for up to 10 h. The floating ability and drug release from the developed devices were governed by the drug release window opening size and the filament material affinity towards the gastric fluid. The designed GRFDs show great prospects in modifying the drug release characteristics and could be applied to any conventional immediate-release product.

## 1. Introduction

The oral route is the most preferred route of drug administration due to high patient compliance, non-invasiveness, low cost, fewer side effects, and better drug absorption profiles achieved by most drug products [1]. However, the conventional oral drug delivery approach may not achieve the desired bioavailability profiles for certain drugs due to physiological limitations of the gastrointestinal tract. Drugs with a narrow absorption window in the upper gastrointestinal tract exhibit low solubility in the alkaline pH of the intestine, with a short biological half-life and are intended for local action in the stomach [2,3]. These drugs require a prolonged gastric residence time in the stomach for absorption. In order to achieve this, several formulation strategies were developed to increase the gastric residence time, which includes mucoadhesive systems, superporous hydrogels, magnetic systems, high-density systems, and gastric floating systems [4,5,6]. Among these, gastric floating systems were preferred as the most simple and practical approach to achieve prolonged gastric retention for gastric drug absorption and local delivery [7]. Moreover, the gastric floating systems do not interfere with the physiological functioning of the gastrointestinal tract [8]. The gastric floating systems are low-density formulations that can float in the gastric media and, thus, prolong the gastric retention time [9]. The gastro-retentive systems were conventionally developed by using microporous polymers, swelling matrices, and gas-generating agents that produce low-density products with an ability to float in the gastric mucosa. The main drawback of this conventional floating drug delivery system development method is its design, which leads to delayed floating lag time, attributing the risk of gastric excretion of the system before producing a floating effect [10]. Thus, the gastric floating system using complex compositions with manufacturing difficulties fails to maintain the dosage form in the stomach for a sufficient time until complete drug release at the pre-determined rate under dynamic physiological conditions [11,12].

Currently, 3D printing technology has been extensively employed for the development of innovative novel drug delivery approaches. Three-dimensional printing techniques can develop complex structures with pre-determined release characteristics with customized dosing for personalized drug delivery [13,14]. Three-dimensional printing is a layer-by-layer deposition technique that builds desired geometries from computer-aided designs using a 3D printer [15,16,17]. With this progress in the application of the 3D printing technique, many researchers fabricated gastric floating systems. Among the various 3D printing techniques, the fused deposition modeling (FDM) technique and pressure-assisted micro-syringe (PAM)-based semisolid extrusion (SSE) technique were extensively used to develop gastric floating systems [16,18]. FDM has led to the development of hollow structures with low infill percentage systems that drives the floating characteristics of the printed floating systems [1,19,20]. The FDM-based 3D printing technique has been employed in two ways to develop gastric floating systems. FDM coupled with the HME technique was used to develop drug-loaded filaments and using this drug-loaded filament, gastric floating systems were designed in different geometries with different infill [21,22,23,24]. In the second FDM-based approach, non-drug-loaded filaments were used to design hollow systems in which the immediate release or sustained release conventional tablets were placed, and by geometrical modification, gastric floating systems were designed [25,26,27,28]. More recently, high drug-loaded gastro-floating tablets of metformin were fabricated by melt extrusion to achieve sustained release robust release kinetics with successful buoyancy [29]. Similarly, a mucoadhesive gastro-retentive matrix tablet for personalized dosing of gabapentin was fabricated by using FDM coupled with the HME technique. The rationale is to overcome absorption and multiple dosing-related drawbacks associated with gabapentin and to obtain maximum buoyancy and sustained drug release [30]. Moreover, FDM 3D printing technique has also been explored to develop mini floating polypills for the personalized treatment of Parkinson’s disease [31]. The PAM technique is used to develop different geometries with low density that imparts floating characteristics to the developed gastric floating systems [32,33,34,35,36]. Recently, a gastro-floating tablet of famotidine was designed by using the SSE-based printing technique. In this approach, a hydroxy propyl methyl cellulose (HPMC)-based hydro-alcoholic gel was prepared to print gastric floating tablets with different infills (10%, 30%, and 50%). The results of this study demonstrated gastric floating and sustained drug release over 10 h with Korsmeyer–Peppas non-Fickian diffusion-based drug release kinetics [37]. Photocurable polymers such as polyethylene glycol diacrylate (PEGDA) have also been used to design metoprolol tartrate gastric floating tablets using an extrusion-based 3D printing technique [38]. The SSE-based 3D printing technique has also been employed to fabricate gastro-retentive drug delivery systems for plant-based natural APIs such as ginkgolides and puerarin [34,39].

The present investigation aims to fabricate a hollow dual-compartment gastro-retentive floating device (GRFD). The outer compartment is an air-filled chamber and the inner compartment is enclosed with an immediate release conventional tablet. In this study, propranolol hydrochloride immediate-release tablets were used to impart floating and modify its release characteristics. Propranolol hydrochloride is a non-selective beta blocker used in the treatment of hypertension and cardiovascular disorders [40,41]. Propranolol hydrochloride is stable in acidic media and exhibits better solubility in an acidic environment (225 mg/mL at 1.2 pH), whereas in an alkaline environment, it is highly unstable and shows a low solubility profile (130 mg/mL at 6.8 pH) [42]. The gastric stability, short elimination half-life, and frequent dosing make propranolol hydrochloride a suitable candidate to fabricate GRFD with prolonged gastric retention time and sustained drug release characteristics. In a previous study, propranolol hydrochloride gastric floating tablets were designed using hot melt extrusion (HME) coupled with the FDM 3D printing technique. This study uses drug-loaded PVA filaments to create ellipsoid-shaped gastric floating tablets with different infill percentages (15% and 25%). The prepared tablets exhibit low floating time (around 2 h) and not as sustained a drug release (>80% drug release in 3 h) [19]. PVA is one of the most commonly employed filament materials in FDM 3D printing due to easy structural formability and hardness [43,44,45]. PVA, due to its hydrophilic nature, tends to result in a shorter gastric floating time. Therefore, to achieve longer gastric floating, PLA, due to its hydrophobicity, was used to fabricate GRFDs [27]. Moreover, PLA is one of the most used polymers for dosage form design by 3D and 4D printing due to its biodegradable nature, required tensile strength, and melting point for the FDM process [46,47]. Our study aims to prolong the gastric floating time and further sustain the drug release. The second objective is to fabricate the gastric floating tablet cost-effectively without using the HME process. Moreover, the use of commercial propranolol tablets in our study makes the process simple and saves the time and effort of designing the conventional immediate-release tablet.

## 2. Materials and Method

### 2.1. Materials

The PVA filament (1.75 mm) was purchased from Fused materials 3D filaments New York, and the PLA filament (1.75 mm) was purchased from FILO3DPRO, made in Holland. Propranolol hydrochloride tablets (Inderal^®^, AstraZeneca, Cambridge, UK) were supplied by Najran University Hospital Pharmacy Najran, Saudi Arabia. Ethanol and hydrochloric acid were purchased from Sigma-Aldrich, Gillingham, UK. All other reagents used were of analytical grade.

### 2.2. Preparation of Gastro-Retentive Floating Device

#### 2.2.1. Selection of Model Drug and Polymer (Filament) for Gastro-Retentive Floating Device

Propranolol Hcl commercial tablets (Inderal^®^, AstraZeneca) were selected as a model drug and incorporated in the designed gastro-retentive floating device. Commercial filaments of PVA and PLA were selected for fabricating gastro-retentive floating devices (GRFD). The selection of these filaments was based on the anti-acidic ability of these materials for 3D printing. Both the filaments of PVA and PLA were immersed IN 0.1 mol/L Hcl solutions and their physical ability to resist the acidic corrosion was observed at different time periods. 

#### 2.2.2. Design of Dual-Compartment Gastro-Retentive Floating Device

**Figure 1:**
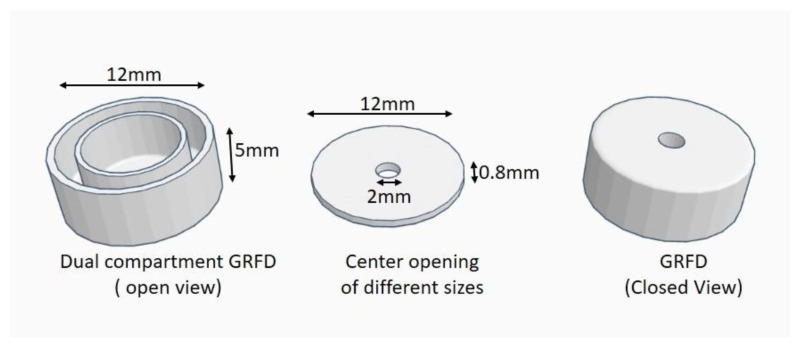
Design and dimensions of the GRFD.

The gastro-retentive floating device (GRFD) was designed as a dual-compartment round tablet of 12 mm diameter and 5 mm thickness with an opening of varying diameters (1 mm, 2 mm, 3 mm, and 4 mm) at the center of the device for drug release. The propranolol Hcl tablets were placed in the inner compartment of the device and the outer compartment was used as an air-filled compartment to facilitate the gastric floating of the device. The design of the GRFD is represented in Figure 1. The GRFD was fabricated by using PVA and PLA commercial filaments. The GRFD was designed by using Autodesk fusion 360 software (version-V.2.0.14335). The fashioned design was saved as an stl file, which was further sliced using flash print flashforge slicer software (Version-5.7.0). The slicer software settings are presented in Table 1. The sliced design was saved as a g-code for 3D printing.

#### 2.2.3. The 3D Printing of Gastro-Retentive Floating Device

The 3D printing of the GRFD was carried out by using an FDM 3D printer (ET Model X1, EASYTHREED Technology CO., Ltd., Shenzhen, China). The pre-dried PVA and PLA filaments were loaded in the extruder of the 3D printer. The 3D printing parameters/conditions are listed in Table 1. GRFD with varying opening sizes was printed by using both PVA and PLA filaments represented in Table 2. The propranolol Hcl tablets were placed in the inner compartment of the GRFD during the 3D printing process before printing the top layer of the GRFD (after 90% of the printing was accomplished). After completing the 3D printing process, the GRFDs were stored in a desiccator for further characterization.

### 2.3. Characterization of 3D-Printed Gastro-Retentive Floating Device

#### 2.3.1. Morphology

Digital images of the GRFD were taken and scanning electron microscopy (SEM) was performed to observe the surface morphology of the GRFD. The height of the layers were measured by using SEM and the presence of gap or flaws between the layers (intactness of the layers) were observed visually and via SEM images.

#### 2.3.2. Dimensions and Weight Variation of the Gastro-Retentive Floating Devices

The size of the GRFD (diameter and thickness) was measured by using a micrometer. The diameter of the center opening in the GRFD was measured by using ImageJ 1.54d software. For weight variation test, twenty GRFDs were weighed individually by using a digital weighing balance (model no. KIA30FKB, Kern & Sohn GmbH, Balingen, Germany) and the weight variation was represented as mean ± SD.

### 2.4. In Vitro Floating Ability

The in vitro floating ability of PVA-based and PLA-based GRFDs was determined by placing them in simulated gastric fluid (0.1 N Hcl, pH 1.2) and the floating lag time (FLT) and total floating time (TFT) were visually determined.

### 2.5. In Vitro Drug Release Characteristics

The in vitro drug release studies were performed for all the floated GRFDs by using USP type II apparatus (Type: DT 820, ERWEKA GmbH D-63150, Heusenstamm, Germany). The dissolution was performed using simulated gastric fluid (0.1 N Hcl, pH 1.2) as media at 37 ± 0.5 °C. A total of 3 mL of aliquots was withdrawn and replaced with the new buffer media to maintain sink conditions. The samples were withdrawn at 5, 15, 30, 45, 60, 120, 180, 240, 300, 360, 420, 480, 540, and 600 min. The withdrawn samples were filtered through a 0.45 µm syringe filtered and analyzed by using a UV spectrophotometer at a wavelength of 290 nm.

### 2.6. Drug Release Kinetics

The in vitro drug release data of GRFDs were fitted to best fit model (zero order, first order, Korsmeyer–Peppas, and Higuchi model) to determine the mechanism of drug release from the GRFDs.

### 2.7. Statistical Analysis

The graphical presentation and statistical analysis were performed using Windows 10 graph pad prism and one-way analysis of variance (ANOVA) followed by Tukey’s test and considered statistically significant when *p* < 0.05. All the results were expressed as mean ± SD.

## 3. Results and Discussion

### 3.1. Preparation of Gastro-Retentive Floating Device

The GRFDs were printed successfully by using PVA and PLA commercial filaments. The aim of fabricating a GRFD is to achieve an extended or prolonged gastric retention time for the device. Based on the observation from the anti-acidic ability of these two filaments between 0 to 3 h, PLA filament tends to resist acidic corrosion and remain rigid even after 3 h. In contrast, the PVA filament tends to become soft and dissolves after 3 h and makes the solution turbid, thus, indicating that PLA-filament-based GRFDs could achieve long-term floating compared to PVA-based GRFDs.

The structural parameter that is altered in the design of GRFD is the diameter or size of the opening. The GRFDs were designed with 1 mm, 2 mm, 3 mm, and 4 mm openings to study the influence of opening size on drug release characteristics. The infill percentage in FDM-based 3D printing varies between 0 to 100%, facilitating the building of hollow structures to filled (densely compact) structures [1,48]. In the design of GRFDs, a low infill percentage (15% infill) was selected to fabricate GRFDs with low density and long gastric floating time.

The printing parameters significantly influence the quality of 3D-printed products [44]. The printing speed, the material flow rate, printing temperature, and the temperature of the bed plate were optimized to print better quality products. The variation in the extrusion rate causes structural deformity. A higher extrusion rate alters the thickness of the product and a lower extrusion rate produces a product of low density but also causes flaws in the top layer printing of the product. Similarly variations in printing temperature also influence the formability of the product. Printing at low temperatures causes non-uniform flow of material and printing at high temperatures causes change in the color of the printed product. The temperature of the printer bed influences the adherence of the product to the bed. Moreover, the surface of the bed plate also influences the adherence of the printed product to the bed depending upon the type of material (filament) used for printing. PLA-based GRFDs were directly printed on the stage of the printer, while printing of PVA-based GRFDs showed adhesion problems of initial layers on the stage of the printer. To overcome this, a brown paper tape was placed on the surface of the printer stage, which facilitated proper adhesion of the printed object. The printing speed represents the speed of material coming out of the nozzle and the travel speed represents the moving speed of the extruder along the *x*, *y*, and *z*-axis [49]. The printing speed and the travel speed have to be optimized relatively or else it may lead to gaps between the layers (inefficient binding between the layers) and rough surfaces of the printed object.) Direct printing was carried out without the raft and brim to reduce the total printing time. The total printing time was approximately 5 min for the printing of each GRFD.

**Figure 2:**
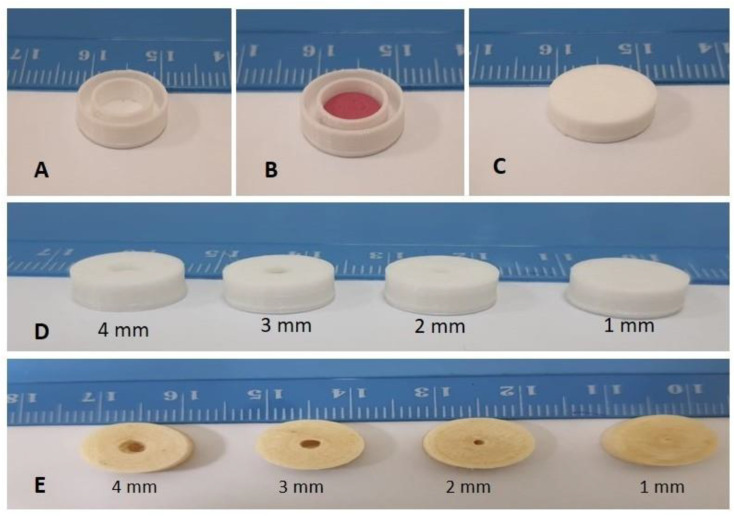
Demonstrating the 3D printing process and fabrication of PLA- and PVA-based GRFDs with different opening sizes [(**A**) open dual compartment GRFD; (**B**) Dual compartment GRFD with tablet; (**C**) closed GRFD; (**D**) PLA based GRFD with different openings (1 mm, 2 mm, 3 mm and 4 mm) at the center of the device (front view); (**E**) PVA based GRFD with different openings (1 mm, 2 mm, 3 mm and 4 mm) at the center of the device (top view)].

The 3D printing of the GRFD was carried out in three steps, as shown in Figure 2. In the first step, more than 90% of the GRFD was printed, which resulted in a dual-shell open GRFD. Secondly, the commercial tablet was placed inside the inner shell of the GRFD and in the third step, the GRFD was sealed. The process of 3D printing of the designed GRFDs comprised 27 layers, made up of an initial 3 solid bottom layers with no gap between them, followed by 2 more layers printed in a rectilinear pattern, then the printing continued until 24 layers, which resulted in more than 90% printing of dual-compartment GRFD. Then, the propranolol Hcl tablet was placed in the inner compartment of the device and the extruder continued to print the top three solid layers to complete the top layers and formed a closed GRFD with different sets of openings at the center (from 1 mm to 4 mm), which facilitated drug release.

### 3.2. Characterization of 3D-Printed Gastro-Retentive Floating Devices

**Figure 3:**
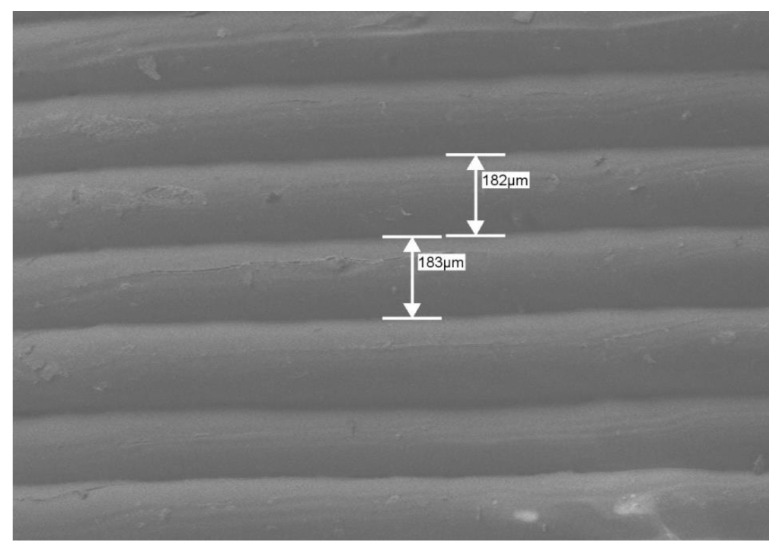
SEM image of the 3D-printed GRFD from the surface view representing layer-by-layer printing pattern.

The morphological characterization of all the PLA- and PVA-based 3D-printed GRFDs indicate that the surface of the GRFDs is smooth with no deformities. The PLA filament is white in color and the PVA filament is slightly yellow in color. The fabricated GRFDs are of the same color as the filament. This is in accordance with the previously reported articles, which state that the 3D printing process fabricates objects of the same color as the feeding material [26,44,50]. The layer height measured via SEM imaging is significant to the printer settings and the layers are found to be intact without any gaps, as represented in Figure 3.

The dimensions of the 3D-printed GRFDs are accurate as they are set in the design represented in Table 3, thus, indicating that the 3D printing technique could produce accurate and uniform-size scaffolds. The weight variation of all the GRFDs is represented in Table 3 as average weight ± SD. The low SD values indicate that 3D printing produces accurate and consistent objects. This result is in accordance with the literature that states the 3D printing process produces tablets with low weight variation [32,44]. The low SD values of weight variation signify consistency in total floating time [51]. The average weight of PVA-based 3D-printed GRFDs is less than the PLA-based GRFDs, which is supported by the pre-printing difference in the initial weights of the two filaments. The pre-printing weight (initial weight before printing) of the PLA and PVA filaments of a fixed length (10 cm) was 305 ± 1.5 mg and 321.5 ± 1.2 mg, respectively. This difference in the initial weights of the filament might be due to the difference in the densities of the materials and their hydrophobic and hydrophilic nature. Thus, this causes a difference in the weight of the printed PLA- and PVA-based GRFDs. The result of the weight variation test reveals that the RSD values for all the GRFDs are less than 5%, which complies with the pharmacopoeial standards. Thus, the FDM 3D printing technique was found to be accurate, and capable of producing products with uniformity and high reproducibility [52]. In the FDM-based 3D printing technique, the variation in the weights of 3D-printed objects is possibly due to variation in filament diameter [53], variation in material flow from the extruder nozzle due to the stop and run process during the printing [54], or due to the removal of support material from the printed object, such as raft and brim.

### 3.3. In Vitro Floating Ability

**Figure 4:**
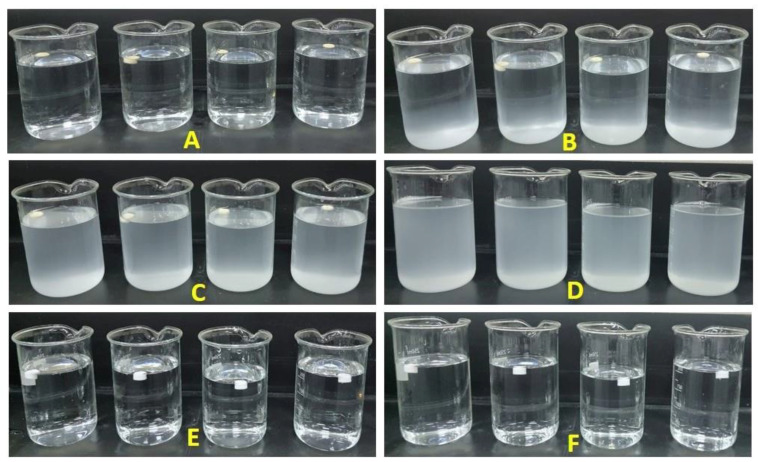
Represents the TFT of the GRFDs fabricated using PVA and PLA filaments ((**A**): GRFD1 to GRFD4 floating at 0 min, (**B**): GRFD1 to GRFD4 floating after 1 h, (**C**): GRFD1 to GRFD4 floating after 2 h, (**D**): GRFD1 to GRFD4 dissolved completely after 3 h, (**E**): GRFD5 to GRFD8 floating at 0 min, and (**F**): GRFD5 to GRFD8 floating after 24 h).

The in vitro floating ability of commercial propranolol Hcl tablets and 3D-printed GRFDs was determined and is represented in Table 4. FLT is the time required by the GRFDs to float. The shorter the FLT, the better the gastric floating attributed to the devices without enduring gastric clearance. The results of FLT indicate that all the printed GRFDs float immediately at 0 min, whereas the propranolol Hcl tablets sank immediately to the bottom. The floating duration is represented as the TFT. The TFT for the GRFDs fabricated from PVA filament (GRFD1 to GRFD4) is more than 3 h. The variation in the TFT exhibited by GRFD1 to GRFD4 is due to the difference in the opening size at the center of the device intended for drug release. The smaller the opening size, the more the TFT exhibited by the device is indicative of low solvent penetration and the slow dissolution of the device. In contrast, the device with a large opening size (4 mm) had greater solvent penetration, and caused the device to dissolve at a faster rate and, thus, reduced the TFT of the device. The floating ability of the printed devices depends upon the air trapped in the device and the total density of the device as indicated in the previous literature [1,55]. The higher the density of the system, the shorter the floating duration. The TFT of the PLA-based GRFDs (GRFD5 to GRFD8) is more than 24 h due to the hydrophobic nature of the PLA, which remains floated without dissolving in the simulated gastric fluid (SGF) without erosion or swelling. The PLA-based devices remain rigid and the opening size at the top of the device does not influence the floating ability of the devices. The GRFDs developed using a 3D printing process show minimum FLT compared to conventionally designed floating drug delivery systems, which prevents the system from undergoing gastric clearance from the stomach without floating [10]. Thus, based on the floating behavior, 3D-printed floating systems exhibit much better floating abilities compared to the conventional methods used to develop these systems [40,42,56]. Figure 4 represents the TFT of the GRFDs fabricated using PVA and PLA filaments. The total floating time achieved by all the GRFDs is more than 3 h, which is ideal as it is longer than the gastric emptying time and, hence, makes them suitable for designing gastro-retentive drug delivery systems.

### 3.4. In Vitro Drug Release Characteristics

**Figure 5:**
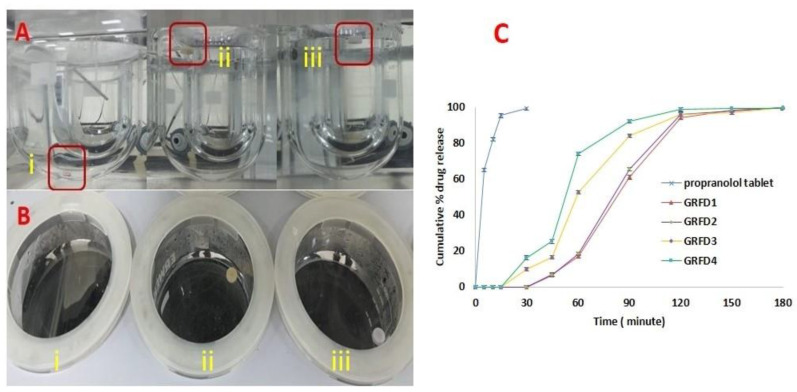
Represents floating behavior of GRFDs and cumulative % drug release of PLA-based GRFDs (**A**): represents the front view of dissolution apparatus with (i) propranolol Hcl tablet sunk, (ii) PVA-based GRFD floating, and (iii) PLA-based GRFD floating; (**B**): represents the top view of dissolution vessel showing (i) propranolol tablet sunk, (ii) PVA-based GRFD floating, and (iii) PLA-based GRFD floating; (**C**): represents cumulative % drug release of immediate-release propranolol tablet and PVA-based GRFDs (GRFD1 to GRFD4).

The in vitro drug release of propranolol Hcl immediate-release tablets and PVA-based GRFDs are shown in Figure 5C. The conventional propranolol immediate-release tablet exhibits >95% of drug release within 20 min, which is in accordance with the reported literature [19,41]. The GRFDs fabricated using PVA and PLA achieve sustained drug release. However, the PVA-based GRFDs (GRFD1 to GRFD4) exhibit a less sustained drug release profile compared to PLA-based GRFDs (GRFD5 to GRFD8) due to their respective affinities towards the SGF. The drug release profile and the lag time for drug release exhibited by GRFDs are opening-size-dependent. The smaller the opening size, the slower the drug release rate and the longer the lag time. Similarly, the larger the opening size, the faster the drug release with a shorter lag time [51,57]. In concordance with this, the GRFD1 and GRFD2 exhibit >60% of drug release in 90 min with a lag time of 30 min, whereas GRFD3 and GRFD4 exhibit >80% of drug release with a lag time of 15 min. This is due to the difference in their drug release window opening size [58]. All the PVA-based GRFDs exhibit >90% of drug release in 2 h. This is due to the hydrophilic nature of the PVA, which results in erosion of the device and the complete release of the enclosed propranolol tablet. To further sustain the drug release from PVA-based GRFDs, strategies that reduce the solubility profile of PVA should be adopted, such as improving the physical properties of PVA by thermal cross-linking, which reduces the solubility of PVA and, thus, provides a sustained drug release of the enclosed tablet [26,59], or by fabricating PVA filaments by adding insoluble excipients. In addition, modifying the GRFD design with varying thicknesses of the shell, cap, and body also prolongs the duration of drug release [60,61].

**Figure 6:**
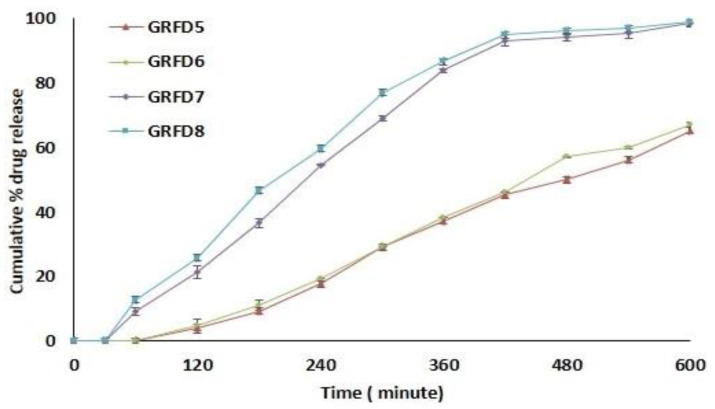
Represents cumulative % drug release from PLA-based GRFDs (GRFD5 to GRFD8).

The PLA-based GRFDs exhibit sustained release of the enclosed propranolol tablet, as shown in Figure 6. GRFD5 and GRFD6 exhibit around 65% of drug release with a lag time of 60 min, whereas GRFD7 and GRFD8 exhibit >95% of drug release with a lag time of 30 min in 10 h duration, as PLA is hydrophobic. Hence, the device remains buoyant and exhibits no swelling, erosion, or eruption. The opening size governs the drug release from these devices. The longer lag time exhibited by these devices is due to the drug release window opening position at the center of the device, which leads to an increased lag time. As the device is floating at the top of the SGF, changing the position of the drug release window to the sides of the device and increasing the number of openings in the device may initiate an immediate release of the drug from the device without the lag time [27,28]. Thus, by designing a PVA-based GRFD by 3D printing, a sustained drug release profile and gastric retention could be achieved similar to commercial sustained release formulations and gastric floating formulations prepared by conventional methods, which, in comparison to 3D printing, requires a wide range of polymers and a series of processing steps [40,62,63]. Thus, 3D printing provides proof of a concept to design a GRFD that provides maximum buoyancy by using PLA without any structural degradation or breakdown in the SGF of the designed system. A study demonstrated that a GRDDS designed by using PLA exhibited an in vivo floating time in rabbits of more than three days due to the hydrophobic nature of the PLA and its slow degradation [27,64,65]. The advantage of the current 3D-printed GRFD is that the floating ability and drug release are independent. The GRFD remains buoyant irrespective of the drug release rate and could remain buoyant even after the complete drug release from the device, whereas in conventional GRFDs, such as raft forming systems, hydrodynamically balanced systems, and gas-generating systems, the floating ability and drug release are dependent and controlled by the floating system itself [66,67]. Any structural changes due to erosion or diffusion mechanism may lead to incomplete buoyancy and the system may undergo gastric emptying without complete drug release. For example, in the conventional GRFD based on a hydrodynamically balanced system, the drug release is controlled by a polymer gel barrier, which also provides buoyancy and the diffusion or erosion of this gel barrier may lead to incomplete buoyancy and early clearing of the system without complete drug release [66]. Thus, the current study provides a simple, cost-effective solution to use any conventional immediate-release tablet in the designed GRFD to achieve delayed and sustained release of the drug. This also reduces the formulation development efforts, cost, and time compared to conventional sustained-release formulation, whose sustained-release performance depends upon drug–polymer interaction.

### 3.5. Drug Release Kinetics

The in vitro drug release data were fitted to the best-fit release kinetic model. The drug release kinetic data of PVA- and PLA-based GRFDs are represented in Table 5. Based on R^2^ values, it is evident that PVA-based GRFD1 and GRFD2, with narrow opening size, exhibit zero-order release kinetics describing a drug release that is independent of the concentration gradient. This is in accordance with the reported literature that reveals that PVA-based gastro-retentive drug delivery systems (GRDDS) exhibit zero-order release kinetics [57,60]. The IR tablet enclosed in the GRFD acts as a barrier for controlling the release. It reduces the enclosed tablet’s exposed surface area, achieving sustained drug release following zero-order release kinetics [27,51]. In comparison, GRFD3 and GRFD4, with wide opening sizes (3 mm and 4 mm), show first-order release kinetics, describing a drug release that is dependent on concentration gradient. Based on the *n* values, the drug release mechanism is diffusion and erosion [19]. Similar results are obtained for PLA-based GRFDs where the drug release is governed by the opening size in the GRFDs. The values of *n* indicates Fickian and non-Fickian-based drug diffusion. All the PLA-based GRFDs exhibit an *n* value greater than 0.5, which indicates non-Fickian-diffusion-based drug release. During the entire drug release time course, the device remains buoyant and does not exhibit any swelling, erosion, or eruption due to the hydrophobic nature of the PLA filament that keeps the device intact, and the drug release is governed by the opening size in the device. Thus, the FDM-based 3D printing technology, due to its low cost, easy processing, and high flexibility, could be utilized to design a product with customized characteristics. This research investigation further extends the progress in the design and development of gastro-retentive drug products.

## 4. Conclusions

The FDM 3D printing technique could produce gastro-retentive floating systems with structural modifications in the design by varying in fill percentage and by producing hollow compartment systems. In conventional methods of producing gastro-retentive floating systems, the polymers and materials play a vital role in achieving buoyancy. These materials or polymers impart buoyancy to the designed floating system by producing effervescence by gas generation or by non-effervescent methods by using low-density materials, by raft-forming, or by producing gel layers. The erosion or dissolution of these polymeric material affects these systems’ floating ability and may lead to buoyancy loss. From the literature, it has been proven that the FDM-based 3D printing has produced a floating device that has floated in vivo for more than three days. This makes this technique the most suitable and efficient method to design gastro-retentive floating systems. In this study, 3D-printed GRFDs were successfully designed by FDM 3D printing using PVA and PLA filaments. These hollow GRFDs enclosed with an immediate release propranolol hydrochloride tablet exhibit efficient buoyancy and sustained drug release profiles. Among the two filament-based devices, the PLA-based GRFDs show maximum buoyancy (>24 h) and sustained drug release (>95% of drug release in 10 h) in comparison to PVA-based GRFDs. The opening in the device (1 mm, 2 mm, 3 mm, and 4 mm) was the critical parameter influencing the drug release rate from the device. The drug release rate increases with an increase in the opening size. Thus, this approach provides an easy, efficient, and cost-effective alternative to conventional methods for designing GRFDs to encapsulate conventional immediate-release tablets to modify their release characteristics. Moreover, the current results show that the structural modification in the GRFDs such as a change in the opening size, number of openings in the device, and their position, affects the buoyancy and drug release rate from the devices. Further in vitro/in vivo correlation studies should be performed to evaluate in vivo floating behavior and drug release profiles. Thus, the FDM-based 3D printing technique enables the researchers to develop patient-tailored drug delivery systems with tunable drug-release characteristics.

## Figures and Tables

**Table 1:** Representing slicing parameters (design settings) and 3D printing parameters (printer settings) used in the fabrication of GRFD.

Slicing Parameters		Printing Parameters	
Parameter	Settings	Parameter	Settings
Layer height	0.18 mm	Filament used—PVA	
First layer height	0.27 mm	Nozzle diameter	0.4 mm
Base print speed	60 mm/s	Filament diameter	1.75 mm
Print speed			
Travel speed	100 mm/s	Printing temperature	200 °C
Shell count	2	Bed temperature	50 °C
Shell thickness	0.8 mm	Filament used—PLA	
Infill density	15%	Nozzle diameter	0.4 mm
Raft	No raft	Filament diameter	1.75 mm
Brim	No brim	Printing temperature	210 °C
Bottom solid layers	3	Bed temperature	40 °C

**Table 2:** Representing various GRFDs designed using PVA and PLA filaments with varying opening sizes (diameter in mm) for drug release.

GRFDs	Filament Used	Opening Size (Diameter in mm)
GRFD1	PVA	1 mm
GRFD2	PVA	2 mm
GRFD3	PVA	3 mm
GRFD4	PVA	4 mm
GRFD5	PLA	1 mm
GRFD6	PLA	2 mm
GRFD7	PLA	3 mm
GRFD8	PLA	4 mm

**Table 3:** Representing dimensions of the actual 3D-printed GRFDs and the average weight of the GRFDs with RSD.

GRFDs	Average Diameter (mm) ± SD	Average Thickness (mm) ± SD	Average Opening Size (mm) ± SD	Average Weight (mg) ± SD	Deviation in Weight (RSD)
GRFD1	12.00 ± 0.02	5.04 ± 0.02	0.11 ± 0.01	432.24 ± 4.24	0.98%
GRFD2	12.02 ± 0.01	5.04 ± 0.01	0.20 ± 0.02	424.30 ± 7.83	1.84%
GRFD3	12.00 ± 0.02	5.03 ± 0.02	0.33 ± 0.02	420.36 ± 8.21	1.95%
GRFD4	12.00 ± 0.02	5.05 ± 0.02	0.41 ± 0.02	418.52 ± 4.36	1.04%
GRFD5	12.03 ± 0.01	5.03 ± 0.03	0.10 ± 0.00	402.42 ± 10.12	2.51%
GRFD6	12.02 ± 0.02	5.02 ± 0.02	0.22 ± 0.08	390.12 ± 5.54	1.42%
GRFD7	12.01 ± 0.01	5.05 ± 0.01	0.31 ± 0.02	382.36 ± 9.76	2.55%
GRFD8	12.00 ± 0.01	5.04 ± 0.03	0.42 ± 0.06	380.46 ± 9.34	2.45%

**Table 4:** Representing density, in vitro floating behavior (TFT and FLT) of propranolol HCl tablet and GRFDs.

	Density (mg/mm^3^)	In Vitro Floating Behavior (*n* = 3)	
		Total Floating Time (h)	Floating Lag Time (min)
Propranolol HCl tablet		0 min	Sunk
GRFD1	0.76 ± 0.01	3 h 42 min 34 s ± 4 min 20 s	0 min
GRFD2	0.75 ± 0.01	3 h 42 min 12 s ± 6 min 16 s	0 min
GRFD3	0.74 ± 0.02	3 h 39 min 54 s ± 7 min 42 s	0 min
GRFD4	0.74 ± 0.01	3 h 33 min 37 s ± 4 min 37 s	0 min
GRFD5	0.71 ± 0.01	>24 h	0 min
GRFD6	0.69 ± 0.01	>24 h	0 min
GRFD7	0.67 ± 0.02	>24 h	0 min
GRFD8	0.67 ± 0.01	>24 h	0 min

**Table 5:** Represents drug release kinetics data of PVA- and PLA-based GRFDs.

GRFDs	Zero-Order (r^2^)	First-Order (r^2^)	Higuchi (r^2^)	Korsmeyer–Peppas	
				(r^2^)	*n* Value
GRFD1	0.9171	0.9028	0.7977	0.5163	0.86
GRFD2	0.9093	0.9057	0.7971	0.5183	0.88
GRFD3	0.8850	0.9218	0.8579	0.3988	0.82
GRFD4	0.8199	0.9392	0.8487	0.3565	0.82
GRFD5	0.9816	0.9567	0.8606	0.8916	0.66
GRFD6	0.9832	0.9564	0.8635	0.8938	0.70
GRFD7	0.9449	0.9497	0.9369	0.8258	0.89
GRFD8	0.9234	0.9623	0.9466	0.7954	0.91

## Data Availability

Not applicable.
